# Crystal structure of *N*-(3-oxo­butano­yl)-l-homoserine lactone

**DOI:** 10.1107/S2056989015024913

**Published:** 2016-01-09

**Authors:** R.W. Newberry, R.T. Raines

**Affiliations:** a Department of Chemistry, University of Wisconsin-Madison, 1101 University Ave., Madison, WI, 53706, USA; b Department of Biochemistry, University of Wisconsin-Madison, 433 Babcock Dr., Madison, WI, 53706, USA

**Keywords:** crystal structure, homoserine lactone, carbonyl inter­action, NBO analysis, hydrogen bonding

## Abstract

The structure and absolute configuration of the title compound, C_8_H_11_NO_4_, which is a known quorum-sensing modulator, have been determined. The mol­ecule exhibits signs of an intra­molecular attractive carbon­yl–carbonyl *n*→π* inter­action between the amide and lactone ester groups, specifically – a short contact of 2.709 (2) Å between the amide oxygen atom and ester carbon atom, approach of the amide oxygen atom to the ester carbonyl group along the Bürgi–Dunitz trajectory, at 99.1 (1)°, and pyramidalization of the ester carbonyl group by 1.1 (1)°. Moreover, a similar *n*→π* inter­action is observed for the amide carbonyl group approached by the ketone oxygen donor. These inter­actions apparently affect the conformation of the uncomplexed mol­ecule, which adopts a different shape when bound to protein receptors. In the crystal, the mol­ecules form translational chains along the *a* axis *via* N—H⋯O hydrogen bonds.

## Abstract 2

This known quorum-sensing modulator exhibits signs of an intra­molecular attractive carbon­yl–carbonyl *n*→π* inter­action between the amide and lactone ester groups. Moreover,a similar *n*→π* inter­action is observed for the amide carbonyl group approached by the ketone oxygen donor. These inter­actions apparently affect the conformation of the uncomplexed mol­ecule, which adopts a different shape when bound to protein receptors.

## Chemical context   

*N*-Acyl homoserine lactones (AHLs) mediate quorum sensing in Gram-negative bacteria (Miller & Bassler, 2001 ▸; Waters & Bassler, 2005 ▸). We have previously shown that AHLs engage in *n*→π* inter­actions between the acyl and lactone ester carbonyl groups (Newberry & Raines, 2014 ▸). These inter­actions cause attraction through donation of oxygen lone pair (*n*) electron density into the π* anti­bonding orbital of an acceptor carbonyl group (Hinderaker & Raines, 2003 ▸). This inter­action is observed in the free mol­ecule but not in structures of these compounds bound to their protein receptors, implicating these inter­actions in the potency of AHLs and their analogs. Background to carbon­yl–carbonyl inter­actions is given by Bretscher *et al.* (2001 ▸), DeRider *et al.* (2002 ▸), Hinderaker & Raines (2003 ▸), and Bartlett *et al.* (2010 ▸). Our previous studies were restricted to AHLs with simple acyl appendages, but natural AHLs are also often oxidized at the 3-position to yield β-keto acyl groups, such as that reported here.
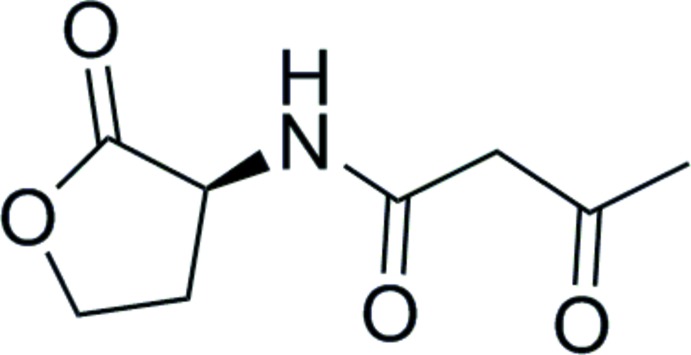


## Structural commentary and NBO analysis   

**Figure 1:**
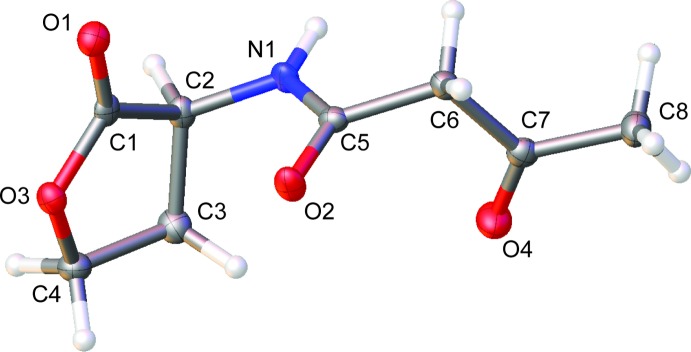
Mol­ecular structure of the title compound with displacement ellipsoids drawn at the 50% probability level.

**Figure 2:**
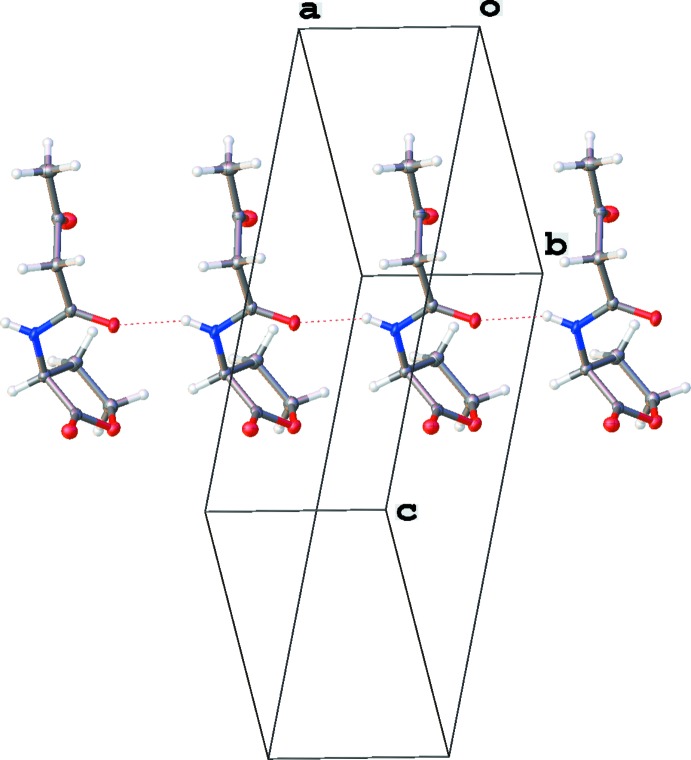
Packing of the title compound.

**Figure 3:**
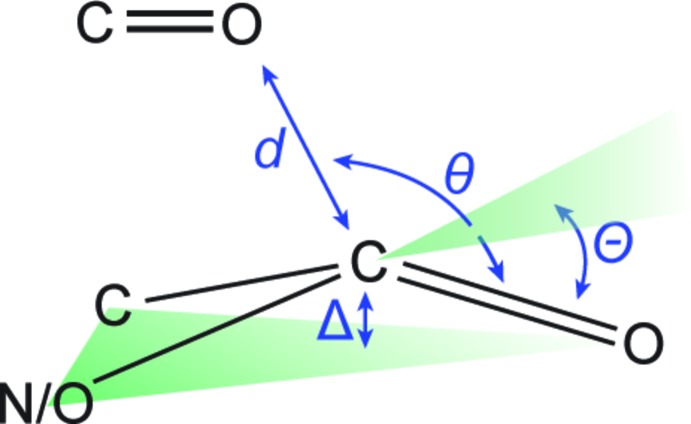
Structural parameters describing an *n*→π* inter­action

**Figure 4:**
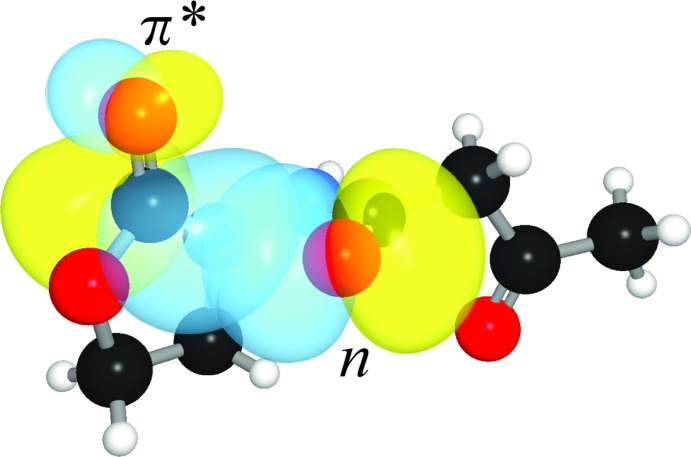
Overlap of amide lone pair (*n*) and ester π* orbitals.

This is, to our knowledge, the first report of the structure of a free 3-oxo AHL (Fig. 1 ▸). Individual mol­ecules pack in linear arrays thanks to inter­molecular hydrogen bonds between amide groups (Fig. 2 ▸). The mol­ecule crystallizes as the keto tautomer, consistent with other β-keto amides (Allen, 2002 ▸). Like unoxidized AHLs, it displays the hallmark features of an attractive *n*→π* inter­action between the amide and ester carbonyl groups (Fig. 3 ▸). Specifically, the donor oxygen atom makes a sub-van der Waals contact of 2.709 (2) Å with the acceptor carbonyl group, with an angle of approach of 99.1 (1)°, characteristic of the Bürgi–Dunitz trajectory for nucleophilic addition (Bürgi *et al.*, 1973 ▸, 1974 ▸). This geometry enables electron donation that, in turn, causes a characteristic pyramidalization of the acceptor carbonyl group. We observe that the carbonyl carbon atom rises 0.016 (1) Å out of the plane of its substituents, creating a distortion angle *θ* (see Fig. 3 ▸) of 1.1 (1)°. This signature has been used to diagnose the presence of these inter­actions in many mol­ecules (Choudhary *et al.*, 2009 ▸, 2014 ▸; Choudhary & Raines, 2011 ▸; Newberry *et al.*, 2013 ▸), including polymers (Newberry & Raines, 2013 ▸) and proteins (Newberry *et al.*, 2014 ▸). Consistent with these observations, natural bond orbital (NBO) analysis (Reed *et al.*, 1988 ▸; Glendening *et al.*, 2012 ▸) of the crystal structure at the B3LYP/6-311+G(2d,p) level of theory predicts the release of 2.67 kcal mol^−1^ of energy due to the *n*→π* inter­action, indicating a significant contribution of this inter­action to the conformation of this mol­ecule (Fig. 4 ▸).

**Figure 5:**
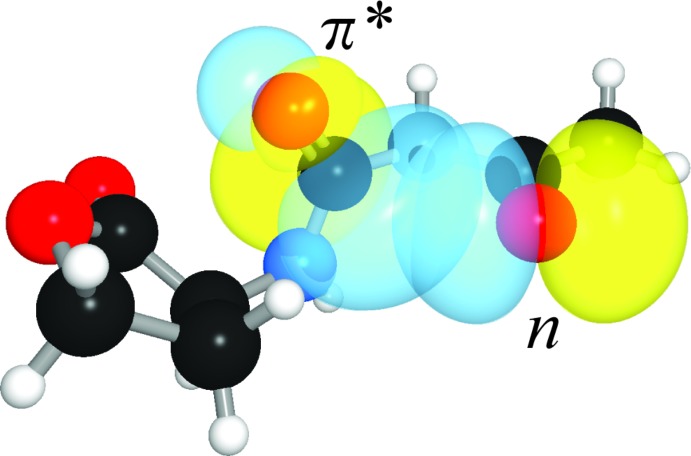
Overlap of ketone lone pair (*n*) and amide π* orbitals.

Inter­estingly, a short contact is also observed between the ketone oxygen and amide carbonyl groups. In this case, the donor oxygen atom makes a 2.746 (2) Å contact at 107.5 (1)° to the amide carbonyl group. This contact causes the amide carbonyl group to distort 0.008 (1) Å out of plane, corresponding to a distortion angle *Θ* of 0.59 (6)°. The pyramidalization of the amide carbonyl group indicates a weaker *n*→π* inter­action from the ketone to the amide than from the amide to the ester, as would be expected for the enclosing of a four-membered ring relative to the enclosing of a five-membered ring, respectively. Indeed, NBO analysis predicts release of 1.42 kcal mol^−1^ of energy due to the *n*→π* inter­action between the ketone and amide (Fig. 5 ▸), which is nevertheless a significant contribution that likely biases the conformation of this mol­ecule.

Based on the specific geometric parameters measured in this crystal structure, we conclude that the structure of unbound oxo-AHLs are influenced by *n*→π* inter­actions, similarly to simple AHLs. Moreover, an additional *n*→π* inter­action specific to oxo-AHLs might bias their conformation further and thus affect their binding to protein receptors.

## Supra­molecular features   

In the crystal, the mol­ecules form translational chains along the a axis *via* N—H⋯O hydrogen bonds (Table 1 ▸ and Fig. 2 ▸).

## Synthesis and crystallization   

The title compound was prepared as reported previously (Eberhard & Schineller, 2000 ▸). A small amount of solid product was dissolved in hexa­nes with a minimal amount of di­chloro­methane. Slow evaporation afforded high-quality crystals after 4 days.

## Refinement   

Crystal data, data collection and structure refinement details are summarized in Table 2 ▸. Except for hydrogen-bond donors and terminal methyl groups, all H atoms were placed in idealized locations and refined as riding with appropriate thermal displacement coefficients *U*_iso_(H) = 1.2 or 1.5 times *U*_eq_(bearing atom).

## Supplementary Material

Crystal structure: contains datablock(s) I. DOI: 10.1107/S2056989015024913/ld2139sup1.cif

Structure factors: contains datablock(s) I. DOI: 10.1107/S2056989015024913/ld2139Isup3.hkl

CCDC reference: 1444720

Additional supporting information:  crystallographic information; 3D view; checkCIF report

## Figures and Tables

**Table 1:** Hydrogen-bond geometry (Å, °)

*D*—H⋯*A*	*D*—H	H⋯*A*	*D*⋯*A*	*D*—H⋯*A*
N1—H1⋯O2^i^	0.83 (2)	2.05 (2)	2.7973 (19)	149 (2)

Symmetry code: (i) 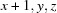.

**Table 2:** Experimental details

Crystal data	
Chemical formula	C_8_H_11_NO_4_
*M*_r_	185.18
Crystal system, space group	Orthorhombic, *P*2_1_2_1_2_1_
Temperature (K)	100
*a*, *b*, *c* (Å)	5.0215 (4), 9.8852 (10), 17.7668 (14)
*V* (Å^3^)	881.91 (14)
*Z*	4
Radiation type	Cu *K*α
μ (mm^−1^)	0.96
Crystal size (mm)	0.23 × 0.13 × 0.04

Data collection	
Diffractometer	Bruker APEXII CCD
Absorption correction	Multi-scan (*SADABS*; Bruker, 2014/5 ▸)
*T*_min_, *T*_max_	0.785, 0.841
No. of measured, independent and observed [*I* > 2σ(*I*)] reflections	11955, 1755, 1702
*R*_int_	0.028
(sin θ/λ)_max_ (Å^−1^)	0.621

Refinement	
*R*[*F* ^2^ > 2σ(*F* ^2^)], *wR*(*F* ^2^), *S*	0.026, 0.067, 1.04
No. of reflections	1755
No. of parameters	134
H-atom treatment	H atoms treated by a mixture of independent and constrained refinement
Δρ_max_, Δρ_min_ (e Å^−3^)	0.22, −0.15
Absolute structure	Flack *x* determined using 657 quotients [(*I* ^+^)−(*I* ^−^)]/[(*I* ^+^)+(*I* ^−^)] (Parsons *et al.*, 2013 ▸).
Absolute structure parameter	−0.01 (8)

Computer programs: *APEX2* (Bruker, 2012 ▸), *SAINT* (Bruker, 2013 ▸), *SHELXS* (Sheldrick, 2008 ▸), *SHELXL* (Sheldrick, 2015 ▸) and *OLEX2* (Dolomanov *et al.*, 2009 ▸).

## supplementary crystallographic information

### Crystal data

**Table:**

C_8_H_11_NO_4_	*D*_x_ = 1.395 Mg m^−^^3^
*M**_r_* = 185.18	Cu *K*α radiation, λ = 1.54178 Å
Orthorhombic, *P*2_1_2_1_2_1_	Cell parameters from 6262 reflections
*a* = 5.0215 (4) Å	θ = 5.0–73.3°
*b* = 9.8852 (10) Å	µ = 0.96 mm^−^^1^
*c* = 17.7668 (14) Å	*T* = 100 K
*V* = 881.91 (14) Å^3^	Block, colourless
*Z* = 4	0.23 × 0.13 × 0.04 mm
*F*(000) = 392	

### Data collection

**Table:**

Bruker APEXII CCD diffractometer	1702 reflections with *I* > 2σ(*I*)
φ and ω scans	*R*_int_ = 0.028
Absorption correction: multi-scan (*SADABS*; Bruker, 2014/5)	θ_max_ = 73.3°, θ_min_ = 5.0°
*T*_min_ = 0.785, *T*_max_ = 0.841	*h* = −6→6
11955 measured reflections	*k* = −12→11
1755 independent reflections	*l* = −22→21

### Refinement

**Table:**

Refinement on *F*^2^	Hydrogen site location: mixed
Least-squares matrix: full	H atoms treated by a mixture of independent and constrained refinement
*R*[*F*^2^ > 2σ(*F*^2^)] = 0.026	*w* = 1/[σ^2^(*F*_o_^2^) + (0.0377*P*)^2^ + 0.2168*P*] where *P* = (*F*_o_^2^ + 2*F*_c_^2^)/3
*wR*(*F*^2^) = 0.067	(Δ/σ)_max_ < 0.001
*S* = 1.04	Δρ_max_ = 0.22 e Å^−^^3^
1755 reflections	Δρ_min_ = −0.15 e Å^−^^3^
134 parameters	Absolute structure: Flack *x* determined using 657 quotients [(*I*^+^)-(*I*^-^)]/[(*I*^+^)+(*I*^-^)] (Parsons *et al.*, 2013).
0 restraints	Absolute structure parameter: −0.01 (8)

### Special details

**Table:**

Geometry. All e.s.d.'s (except the e.s.d. in the dihedral angle between two l.s. planes) are estimated using the full covariance matrix. The cell e.s.d.'s are taken into account individually in the estimation of e.s.d.'s in distances, angles and torsion angles; correlations between e.s.d.'s in cell parameters are only used when they are defined by crystal symmetry. An approximate (isotropic) treatment of cell e.s.d.'s is used for estimating e.s.d.'s involving l.s. planes.

### Fractional atomic coordinates and isotropic or equivalent isotropic displacement parameters (Å^2^)

**Table:**

	*x*	*y*	*z*	*U*_iso_*/*U*_eq_	
O1	0.1639 (3)	0.52850 (12)	0.55760 (7)	0.0190 (3)	
O2	−0.0589 (2)	0.37968 (12)	0.41512 (7)	0.0189 (3)	
N1	0.3857 (3)	0.39964 (14)	0.42086 (8)	0.0156 (3)	
O3	0.0157 (2)	0.68079 (12)	0.47556 (7)	0.0164 (3)	
O4	0.2366 (3)	0.25259 (13)	0.26283 (7)	0.0253 (3)	
C4	0.0901 (4)	0.73341 (18)	0.40163 (10)	0.0189 (4)	
H4A	0.1855	0.8206	0.4069	0.023*	
H4B	−0.0703	0.7480	0.3703	0.023*	
C7	0.2289 (3)	0.15843 (17)	0.30622 (9)	0.0166 (3)	
C1	0.1762 (3)	0.57897 (16)	0.49600 (9)	0.0141 (3)	
C8	0.2475 (5)	0.01346 (18)	0.28126 (11)	0.0230 (4)	
C5	0.1638 (3)	0.32746 (17)	0.41024 (9)	0.0142 (3)	
C6	0.2005 (3)	0.17999 (16)	0.39064 (9)	0.0161 (3)	
H6A	0.0454	0.1279	0.4092	0.019*	
H6B	0.3615	0.1451	0.4163	0.019*	
C2	0.3719 (3)	0.54444 (16)	0.43286 (10)	0.0158 (3)	
H2	0.5528	0.5780	0.4472	0.019*	
C3	0.2703 (4)	0.62767 (17)	0.36590 (10)	0.0200 (4)	
H3A	0.4199	0.6710	0.3387	0.024*	
H3B	0.1696	0.5701	0.3303	0.024*	
H1	0.534 (5)	0.363 (2)	0.4159 (12)	0.018 (5)*	
H8A	0.389 (5)	−0.031 (3)	0.3095 (14)	0.030 (6)*	
H8B	0.073 (6)	−0.032 (3)	0.2945 (15)	0.044 (8)*	
H8C	0.272 (6)	0.006 (3)	0.2277 (15)	0.034 (6)*	

### Atomic displacement parameters (Å^2^)

**Table:**

	*U*^11^	*U*^22^	*U*^33^	*U*^12^	*U*^13^	*U*^23^
O1	0.0201 (6)	0.0182 (6)	0.0187 (6)	−0.0031 (5)	0.0022 (5)	0.0006 (5)
O2	0.0105 (5)	0.0182 (6)	0.0279 (6)	0.0009 (5)	0.0000 (5)	−0.0035 (5)
N1	0.0093 (6)	0.0163 (7)	0.0213 (7)	0.0032 (6)	0.0009 (5)	−0.0034 (6)
O3	0.0141 (5)	0.0160 (6)	0.0192 (6)	0.0013 (5)	0.0027 (5)	−0.0007 (5)
O4	0.0359 (8)	0.0200 (6)	0.0201 (6)	0.0004 (6)	0.0007 (6)	0.0030 (5)
C4	0.0192 (8)	0.0199 (8)	0.0176 (8)	0.0009 (7)	−0.0012 (7)	0.0018 (7)
C7	0.0131 (7)	0.0182 (8)	0.0185 (8)	−0.0006 (7)	−0.0008 (6)	0.0003 (6)
C1	0.0107 (7)	0.0125 (7)	0.0191 (8)	−0.0046 (6)	0.0004 (6)	−0.0033 (6)
C8	0.0316 (10)	0.0185 (8)	0.0190 (8)	0.0005 (8)	−0.0004 (8)	−0.0024 (7)
C5	0.0125 (7)	0.0170 (7)	0.0132 (7)	0.0016 (7)	0.0000 (6)	0.0009 (6)
C6	0.0158 (8)	0.0145 (7)	0.0180 (8)	0.0011 (7)	−0.0001 (6)	0.0005 (6)
C2	0.0121 (7)	0.0155 (8)	0.0196 (8)	−0.0013 (6)	0.0021 (6)	−0.0028 (6)
C3	0.0205 (8)	0.0202 (8)	0.0193 (8)	−0.0001 (8)	0.0037 (7)	0.0012 (6)

### Geometric parameters (Å, º)

**Table:**

O1—C1	1.204 (2)	C2—C3	1.534 (2)
O2—C5	1.235 (2)	C2—H2	1.000
N1—C5	1.337 (2)	C3—H3a	0.990
N1—C2	1.449 (2)	C3—H3b	0.990
O3—C4	1.461 (2)	C4—H4a	0.990
O3—C1	1.340 (2)	C4—H4b	0.990
O4—C7	1.209 (2)	N1—H1	0.83 (2)
C4—C3	1.521 (2)	C6—H6a	0.990
C7—C8	1.503 (2)	C6—H6b	0.990
C7—C6	1.522 (2)	C8—H8a	0.98 (3)
C1—C2	1.530 (2)	C8—H8b	1.01 (3)
C5—C6	1.510 (2)	C8—H8c	0.96 (3)
			
C5—N1—C2	120.55 (14)	C4—C3—H3a	111.0
C1—O3—C4	110.93 (13)	C4—C3—H3b	111.0
O3—C4—C3	106.42 (13)	H3a—C3—H3b	109.0
O4—C7—C8	122.95 (15)	C3—C4—H4a	110.4
O4—C7—C6	121.57 (15)	C3—C4—H4b	110.4
C8—C7—C6	115.48 (14)	O3—C4—H4a	110.4
O1—C1—O3	121.79 (15)	O3—C4—H4b	110.4
O1—C1—C2	127.35 (15)	H4a—C4—H4b	108.6
O3—C1—C2	110.82 (14)	C2—N1—H1	119.2 (15)
O2—C5—N1	121.47 (15)	C5—N1—H1	119.9 (15)
O2—C5—C6	122.02 (15)	C5—C6—H6a	109.2
N1—C5—C6	116.50 (14)	C5—C6—H6b	109.2
C5—C6—C7	111.96 (13)	C7—C6—H6a	109.2
N1—C2—C1	111.04 (13)	C7—C6—H6b	109.2
N1—C2—C3	115.58 (15)	H6a—C6—H6b	107.9
C1—C2—C3	103.61 (14)	C7—C8—H8a	108.9 (17)
C4—C3—C2	104.05 (14)	C7—C8—H8b	107.5 (17)
C1—C2—H2	108.8	C7—C8—H8c	111.9 (18)
N1—C2—H2	108.8	H8a—C8—H8b	108 (2)
C3—C2—H2	108.8	H8b—C8—H8c	108 (2)
C2—C3—H3a	111.0	H8c—C8—H8a	112 (2)
C2—C3—H3b	111.0		

### Hydrogen-bond geometry (Å, º)

**Table:**

*D*—H···*A*	*D*—H	H···*A*	*D*···*A*	*D*—H···*A*
N1—H1···O2^i^	0.83 (2)	2.05 (2)	2.7973 (19)	149 (2)

Symmetry code: (i) *x*+1, *y*, *z*.
